# Screening ionic liquids for developing advanced immobilization technology for CO_2_ separation

**DOI:** 10.3389/fchem.2022.941352

**Published:** 2022-07-12

**Authors:** Zhengxing Dai, Yifeng Chen, Yunhao Sun, Zhida Zuo, Xiaohua Lu, Xiaoyan Ji

**Affiliations:** ^1^ Energy Engineering, Division of Energy Science, Luleå University of Technology, Luleå, Sweden; ^2^ State Key Laboratory of Material-Oriented Chemical Engineering, Nanjing Tech University, Nanjing, China

**Keywords:** CO2 separation, ePC-SAFT, ionic liquids, immobilization, compressibility

## Abstract

Developing immobilized-ionic liquids (ILs) sorbents is important for CO_2_ separation, and prior theoretically screening ILs is desirable considering the huge number of ILs. In this study, the compressibility of ILs was proposed as a new and additional index for screening ILs, and the developed predictive theoretical model, i.e., electrolyte perturbed-chain statistical associating fluid theory, was used to predict the properties for a wide variety of ILs in a wide temperature and pressure range to provide systematic data. In screening, firstly, the isothermal compressibilities of 272 ILs were predicted at pressures ranging from 1 to 6,000 bar and temperatures ranging from 298.15 to 323.15 K, and then 30 ILs were initially screened. Subsequently, the CO_2_ absorption capacities in these 30 ILs at temperatures from 298.15 to 323.15 K and pressures up to 50 bar were predicted, and 7 ILs were identified. In addition, the CO_2_ desorption enthalpies in these 7 ILs were estimated for further consideration. The performance of one of the screened ILs was verified with the data determined experimentally, evidencing that the screen is reasonable, and the consideration of IL-compressibility is essential when screening ILs for the immobilized-IL sorbents.

## Introduction

Excessive CO_2_ emissions have led to serious problems and received great concern (Figueres et al., 2018). According to the report from International Energy Agency, the amount of CO_2_ emissions in 2020 was already around 30 Gt (Iea, 2020), and the excessive CO_2_ emissions have led to environmental problems, such as global warming, glacial melting, and seawater acidification (Clark et al., 2020; Singh and Polvani, 2020; Hanna et al., 2021). To reduce CO_2_ emissions, carbon capture and storage (CCS) has been proposed as one of the important options, in which CO_2_ separation is often needed to capture CO_2_. Technologies have been developed for CO_2_ separation, which can be divided into four categories, absorption, adsorption, membrane, and cryogenic (D'Alessandro et al., 2010), while the energy usage and cost of these traditional technologies are still high (Figueroa et al., 2008). Hence, developing energy-efficient and cost-effective technology for CO_2_ separation is necessary, and novel absorbent development is one of the research focuses.

Ionic liquids (ILs) are molten salts with cation and anion as constituents while in the liquid state at room temperature (Zhang et al., 2017; Chen et al., 2020). ILs have the advantages of low vapor pressure, high thermal stability, and designable ability. Some ILs possess relatively high CO_2_ solubility and selectivity over other gases (e.g., N_2_ and CH_4_) as well as low regeneration temperature and desorption enthalpy, making them desirable absorbents for CO_2_ separation (Zhang et al., 2012; Wang et al., 2020). Many ILs have been designed and synthesized for CO_2_ separation, such as pyrrolidinium-, imidazolium-, quaternary ammonium-, and quaternary phosphonium-based ILs (Blanchard et al., 2001; Anderson et al., 2007; Kilaru et al., 2007). However, their high cost and high viscosity (i.e., low CO_2_ mass transfer rate) are still the current drawbacks hindering industrial applications of ILs on a large scale (Goodrich et al., 2010; Li et al., 2013).

Immobilizing ILs on porous materials is an effective strategy to overcome the above deficiencies (Yan et al., 2011). Zhang et al. found that the CO_2_ absorption rate in the tetrabutylphosphonium amino acid salts immobilized on silica (SiO_2_) was much higher than that in the bulk phase (Zhang et al., 2006). Wang et al. immobilized 1-ethyl-3-methylimidazolium amino acid on the surface of the polymethylmethacrylate microspheres. It was found that the CO_2_ sorption equilibrium could be reached within 15 min (Wang et al., 2013b), which is also much faster compared to that in the bulk phase. It is widely accepted that the intensification of CO_2_ absorption rate is owing to the large mass transfer area after IL immobilization (Zhang et al., 2009; Vicent-Luna et al., 2013; Wang et al., 2013a; Khan et al., 2014). However, the abnormally high CO_2_ absorption capacity in the immobilized ILs was also observed. For instance, Zhang et al. noticed that the CO_2_ absorption capacity in the 1-butyl-3- methylimidazolium tetrafluoroborate [(C_4_mim) (BF_4_)] confined in mesoporous silica gels was improved by about 1.5 times (Zhang et al., 2010). Wu et al. observed that the CO_2_ absorption capacity of 1-hexyl-3-methylimidazolium bis((trifluoromethyl)sulfonyl)imide ([C_6_mim][Tf_2_N]) was increased from 0.031 to 0.386 mol-CO_2_/mol-IL after immobilized on the surface of titanium dioxide (Wu et al., 2017). Therefore, the enhanced CO_2_ absorption capacity can be another important reason to improve the CO_2_ absorption performance, and immobilizing ILs on the porous materials is a promising way to promote the development of IL-based technologies for CO_2_ separation.

To develop IL-immobilized sorbent for CO_2_ separation, it is desirable to make a prior theoretical screening based on the properties of ILs, owing to the huge amount of ILs (up to 10^18^) that can be potentially synthesized. Normally, CO_2_ absorption capacity and selectivity as well as desorption enthalpy are considered to evaluate the CO_2_ separation performance. Among them, CO_2_ absorption capacity directly shows the ability of ILs to capture CO_2_, and desorption enthalpy reflects the energy usage for regeneration. Consequently, these two properties can be used to primarily evaluate the performance of ILs and are often used as the index in IL screening (Maiti, 2009; Palomar et al., 2011; Lee and Lin, 2015; Zhang et al., 2016; Taheri et al., 2021), which is valid for the technologies where the bulk ILs are used. While as discussed in the previous paragraph, when ILs are immobilized, the IL properties will change, causing a difference in CO_2_ absorption capacity from its bulk. For example, based on molecular simulations, Pinilla et al. found that the density of 1,3-dimethylimidazolium chloride in a confined space is twice that in the bulk phase (Pinilla et al., 2005), Sha et al. observed a liquid-to-solid phase transition monolayer when 1,3-dimethylimidazolium chloride was confined between the graphite walls (Sha et al., 2008) and confirmed its higher melting point (Sha et al., 2009). This evidenced that when IL is immobilized, due to the asymmetric and strong interaction between the IL molecule and solid surface, the properties of ILs may be very different from its bulk phase, which need to be considered in screening immobilized ILs for CO_2_ separation.

To consider the special properties of the immobilized ILs, the quantity related to the density change can be used as an additional index. Both advanced experiments and computer simulations have evidenced a higher density of the immobilized ILs compared to the bulk (Shi and Sorescu, 2010; Shi and Luebke, 2013). In particular, as reported by Gubbins et al., the molecules in a fluid or solid film adsorbed on a solid substrate experience strong compression, which is equivalent to a pressure up to several thousand bar (Gubbins et al., 2018). Based on these observations, it can be inferred that the enhanced CO_2_ absorption capacity in the immobilized ILs comes from the complex interaction between the IL and substrate, which may be reflected by the density change. In other words, the compressibility of ILs may be an essential factor in determining the CO_2_ absorption capacity, i.e., the higher the compressibility, the higher the potential to pressurize IL (via the interaction between IL and substrate) to increase density, and the higher the CO_2_ absorption capacity due to the increased density. Therefore, besides the CO_2_ absorption capacity and desorption enthalpy, the compressibility of IL at the pressure that is equivalent to the asymmetric and strong interaction with the substrate, might be an additional index in screening ILs when developing immobilized-ILs for CO_2_ separation.

Due to the equivalent pressure is extremely high (up to several thousand bar), it is hard to determine the compressibility of ILs by using experimental measurements, and thus model prediction can be a viable option. A lot of theoretical models have been developed to predict the properties of ILs, which can be used as theoretical tools to screen ILs, such as Conductor-like Screening Model for Real Solvents (COSMO-RS), COSMO segment activity coefficient model (COSMO-SAC), Soave Redlich Kwong, Universal Quasi–Chemical Model, and so on (Maiti, 2009; Gonzalez-Miquel et al., 2011; Lee and Lin, 2015; Farahipour et al., 2016; Kamgar and Rahimpour, 2016; Theo et al., 2016). However, none of them can be used at high pressures. In our previous work (Ji and Adidharma, 2008; Ji and Adidharma, 2010; Ji et al., 2012; Shen et al., 2015; Ji and Held, 2016; Shen et al., 2018; Sun et al., 2020), electrolyte perturbed-chain statistical associating fluid theory (ePC-SAFT) has been developed with ion-specific parameters, and, in particular, the model can be used up to high pressures owing to the consideration of the dispersion between IL-cations and IL-anions. The model performance has been verified extensively (Ji and Adidharma, 2012; Ji et al., 2013; Ji et al., 2014; Shen et al., 2015; Bülow et al., 2019; Sun et al., 2020; Sun et al., 2021). All these make ePC-SAFT a powerful tool for predicting the compressibility of ILs in a wide pressure range.

In this work, for the first time, the compressibility of IL at high pressures was proposed as a new index, which was then combined with the CO_2_ absorption capacity and desorption enthalpy to screen ILs for developing immobilized-ILs for CO_2_ separation. The ePC-SAFT model was used to predict the properties in a wide temperature and pressure range to provide systematic data for screening ILs step by step. In addition, to verify the screening results, the CO_2_ separation performance of the screened ILs was compared with the experiments.

## Theory

### ePC-SAFT

ePC-SAFT was developed by Cameretti and Sadowski (Cameretti et al., 2005), as an extension of PC-SAFT proposed by Gross and Sadowski (Gross and Sadowski, 2000; Gross and Sadowski, 2001). In ePC-SAFT, the dimensionless residual Helmholtz energy (*A* ^*res*^) is expressed as

$$A^{\mathrm{res}}=A^{\mathrm{hc}}+A^{\mathrm{disp}}+A^{\mathrm{ion}}$$ (1)

Where *A* ^*hc*^ and *A* ^*disp*^ are the contributions from the hard chain and dispersive terms, respectively, and the expressions can be obtained from the literature (Gross and Sadowski, 2001). The ionic term ( $A^{ion}$ ) was represented by the Debye-Hückel theory (Gross and Sadowski, 2001)

$$A^{ion}=-\frac{\kappa }{12\pi \varepsilon_{\tau }\varepsilon_{0}}\cdot \underset{j}{\sum }x_{j}q_{j}^{2}\chi_{j}$$ (2)

Where *κ* is the inverse Debye-screening length with a unit of reciprocal meter, $\varepsilon_{\tau }$ is the relative dielectric constant of the medium, $\varepsilon_{0}$ is the dielectric constant of vacuum, $x_{j}$ is the mole fraction of ion *j*, and $q_{j}$ is the charge of ion *j*. The units of $\varepsilon_{0}$ , *κ*, and $q_{j}$ are *F*/*m*, reciprocal meter, and coulomb, respectively. The definitions of *κ* and $\chi_{j}$ have been described in detail in the original ePC-SAFT (Gross and Sadowski, 2001).

In 2012, ePC-SAFT was extended to predict the properties of ILs, where each IL was assumed to be fully dissociated into IL-anion and IL-cation (Ji et al., 2012). Each IL-ion with three parameters, i.e., segment number, the segment diameter, and the segment energy, while $\varepsilon_{\tau }$ was set to be unity for pure ILs. In particular, dispersive interaction exists between IL-cation and IL-anion, which is different from the ordinary aqueous electrolyte solutions.

In modeling, the parameters of ePC-SAFT for each IL-ion were taken from the literature, which were fitted to the experimental liquid-density of pure ILs or estimated with the linear equation based on the molar weight of IL-ions (Ji and Held, 2016).

Following ePC-SAFT, the density can be obtained from the dimensionless residual Helmholtz energy numerically at different temperatures and pressures, and then other thermodynamic properties can be further derived, such as compressibility, fugacity coefficient, etc. The combination of thermodynamic properties and phase equilibria can be used to predict the gas solubility, such as CO_2_ solubility in ILs, and the relevant properties, such as desorption enthalpy, can be further obtained.

### Compressibility

Following ePC-SAFT, the isothermal compression coefficient ( $\kappa_{T}$ ) of ILs can be estimated with Eqn. 3:

$$\kappa_{T}^{-1}=\rho {\left(\frac{\partial P}{\partial \rho }\right)}_{T}$$ (3)

Where *P* is the pressure in bar, *T* is the temperature in Kalvin, and ρ is the density of ILs obtained from Eqn. 4.

$$P=\rho^{2}{\left(\frac{\partial A^{res}}{\partial \rho }\right)}_{T}$$ (4)

### CO_2_ solubility

Following our previous study (Ji et al., 2012), the vapor pressure of ILs is negligible, and the phase equilibrium for CO_2_ in a CO_2_-IL system can be represented by the following equation:

$$\varphi_{CO_{2}}^{V}\left(T,v^{V}\right)=x_{CO_{2}}\cdot \varphi_{CO_{2}}^{L}\left(T,v^{L},x_{CO_{2}}\right)$$ (5)

Where $x_{CO_{2}}$ is the mole fraction of CO_2_ in the liquid phase, $\varphi_{CO_{2}}^{L}$ and $\varphi_{CO_{2}}^{V}$ are the fugacity coefficients for CO_2_ in the liquid and vapor phases, respectively, and $v^{L}$ and $v^{V}$ are the molar volumes of liquid and vapor phases, respectively. In this work, $\varphi_{CO_{2}}^{L}$ , $\varphi_{CO_{2}}^{V}$ , $v^{L}$ , and $v^{V}$ were calculated with ePC-SAFT, where the parameters of CO_2_ were taken from the original PC-SAFT (Gross and Sadowski, 2001).

### Desorption enthalpy

In this work, the ILs that physically absorb CO_2_ were considered, and thus, the CO_2_ desorption enthalpy (Δ*H*) can be calculated with the following equation:

$$\Delta H=R\left(\frac{\partial lnH_{CO_{2}}\left(T\right)}{\partial \left(1/T\right)}\right)$$ (6)

where $H_{CO_{2}}\left(T\right)$ is the Henry’s constant of CO_2_ in the IL. In this work, the value of $H_{CO_{2}}\left(T\right)$ was calculated with Eqn. 7

$$H_{CO_{2}}\left(T\right)=\underset{x\to 0}{lim}\frac{P\varphi_{CO_{2}}^{V}}{x_{CO_{2}}}$$ (7)

In Eqn. 7, the fugacity coefficient ( $\varphi_{CO_{2}}^{V}$ ) of CO_2_ in the vapor phase was calculated with Eqs. 8–10:

$$ln\varphi_{CO_{2}}^{V}=\frac{\mu_{CO_{2}}^{res}\left(T,v^{V}\right)}{kT}-lnZ$$ (8)

$$\frac{\mu_{CO_{2}}^{res}\left(T,V\right)}{kT}=A^{res}+\left(Z-1\right)+{\left(\frac{\partial A^{res}}{\partial x_{CO_{2}}}\right)}_{T,V}-x_{CO_{2}}{\left(\frac{\partial A^{res}}{\partial x_{CO_{2}}}\right)}_{T,V}$$ (9)

$$Z=Pv^{V}/RT$$ (10)

Where, $\mu_{CO_{2}}^{res}\left(T,v^{V}\right)$ is the chemical potential of CO_2_, *k* is the Boltzmann constant (1.380649 × 10^–23^ J/K), *Z* is the compressibility factor, and *R* is the gas constant [8.314J/ (mol·K)].

## Results and discussion

To screen ILs for developing IL-immobilization technology, in this work, it was achieved step by step. Firstly, compressibility was used to reflect the potential in density increase for enhancing the CO_2_ absorption, and then ILs were primarily screened. Subsequently, the ILs with high compressibility were further screened based on the CO_2_ absorption capacity. Additionally, the desorption enthalpy of the screened ILs was predicted for further verification. Finally, the screened ILs were verified with the available experimental data.

The screening is based on the properties predicted theoretically with ePC-SAFT. In predicting compressibility, to represent the interaction between the IL molecule and solid surface, we set the pressures ranging from 1 to 6,000 bar. Generally, for a CO_2_ separation process, the absorption can be from room temperature to 313.15 K. Considering the heat release during absorption, the temperature was set from 298.15 to 323.15 K. Meanwhile, according to the applicability of ePC-SAFT and practical applications, the conditions in predicting the CO_2_ absorption capacity and desorption enthalpy were set to be 298.15 to 323.15 K and 1 to 50 bar.

### Compressibility

The studied ILs contain the IL-cations of [C_n_mim]^+^, [C_n_py]^+^, [C_n_mpy]^+^, [C_n_mpyr]^+^, and [THTDP]^+^, and the IL-anions of [Tf_2_N]^-^, [PF_6_]^-^, [BF_4_]^-^, [tfo]^-^, [DCA]^-^, [SCN]^-^, [C_1_SO_4_]^-^, [C_2_SO_4_]^-^, [eFAP]^-^, Cl^−^, [Ac]^-^, and Br^−^.

**FIGURE 1:**
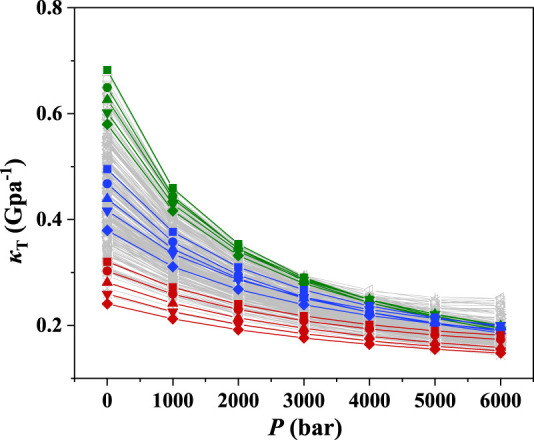
*κ*_T_ of 272 ILs predicted with ePC-SAFT at 298.15 K.

The ePC-SAFT model with the available parameters was used to predict the isothermal compression coefficient of ILs. The results at 298.15 K and 1–6,000 bar were illustrated as an example as depicted in Figure 1. The green lines in Figure 1 represent the ILs with good compressibility, while the red ones indicate the ILs with poor compressibility. As shown in Figure 1, the compressibility of ILs can be significantly different, and the highest compressibility is around 3 times of the lowest one at atmospheric pressure. The IL compressibility with the significant difference makes it essential to consider the compressibility when screening ILs for developing the IL-immobilized absorbent.

**FIGURE 2:**
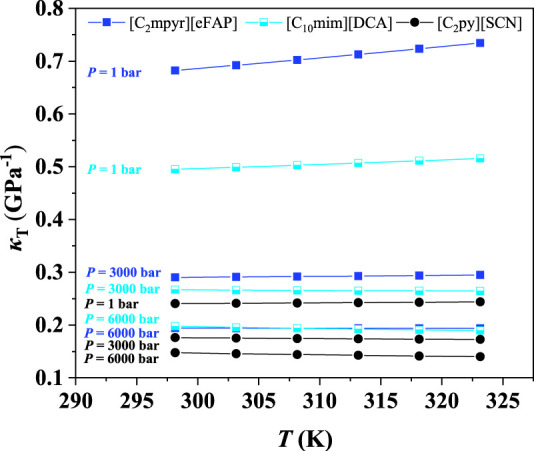
*κ*_T_ of ILs at different temperatures.

Furthermore, it was found that the isothermal compression coefficient decreases with increasing pressure, indicating that it becomes more difficult to further increase the IL density at high pressures. The same phenomena were observed at other temperatures, as depicted in Supplementary Figures S1–S5. The IL compressibilities at different temperatures are listed in Supplementary Table S1. To further illustrate the effect of temperature on the compressibility, [C_2_py][SCN], [C_10_mim][DCA], and [C_2_mpyr][eFAP], which represent the ILs with poor, medium, and good compressibility, respectively, were selected as three representatives. The results are shown in Figure 2, evidencing that the compressibility is slightly affected by temperature. Other ILs also showed the same phenomena.

**FIGURE 3:**
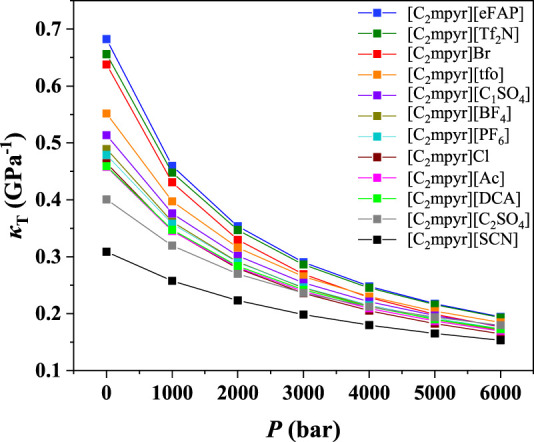
*κ*_T_ value of ILs with [C_2_mpyr]^+^.

To illustrate the influences of anions and cations, the compressibilities of [C_2_mpyr]^+^, [C_2_mpy]^+^, [C_2_mim]^+^, [C_2_py]^+^ and [THTDP]^+^ at 298.15 K were further analyzed as examples to illustrate the effect of anions. The results are depicted in Figure 3 and Supplementary Figures S6–S9. As shown in Figure 3, it was found that the compressibility of ILs with the cation of [C_2_mpyr]^+^ decreased with the order of [eFAP]^-^ > [Tf_2_N]^-^ > Br^−^ > [tfo]^-^ > [C_1_SO_4_]^-^ > [BF_4_]^-^ > [PF_6_]^-^ > Cl^−^ > [DCA]^-^ > Ac^−^ > [C_2_SO_4_]^-^ > [SCN]^-^. For the other cations, the effect of anions is quite similar to that of [C_2_mpyr]^+^. Therefore, it can be inferred that the anions play the important role on the compressibility of ILs.

**FIGURE 4:**
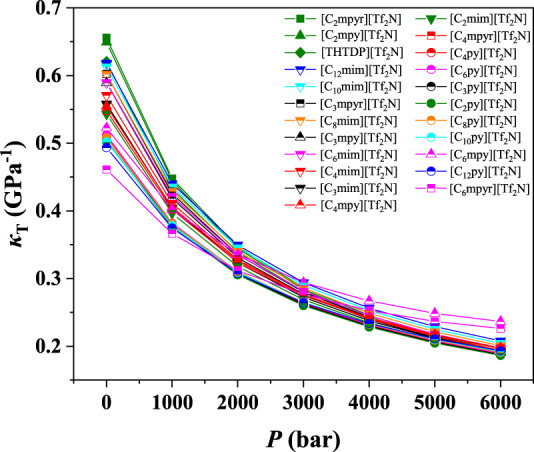
*κ*_T_ value of ILs with [Tf_2_N]^-^.

To study the influence of cations on the compressibility of ILs, [eFAP]^-^, [Tf_2_N]^-^, [BF_4_]^-^, [PF_6_]^-^, [C_2_SO_4_]^-^, and [SCN]^-^ were selected for investigation. The results of their isothermal compressibilities are shown in Figure 4 and Supplementary Figures S10–S14. As depicted in Figure 4, for the ILs with the cation of [Tf_2_N]^-^, the compressibility of ILs follows the order of [C_2_mpyr]^+^ > [C_2_mpy]^+^ > [THTDP]^+^ > [C_12_mim]^+^ > [C_10_mim]^+^ > [C_3_mpyr]^+^ > [C_8_mim]^+^ > [C_6_mim]^+^ > [C_3_mpy]^+^ > [C_4_mim]^+^ > [C_3_mim]^+^ > [C_4_mpy]^+^ > [C_4_mpyr]^+^ > [C_2_mim]^+^ > [C_6_mpy]^+^ > [C_6_py]^+^ > [C_4_py]^+^ > [C_8_py]^+^ > [C_3_py]^+^ > [C_2_py]^+^ > [C_10_py]^+^ > [C_12_py]^+^ > [C_6_mpyr]^+^. While for the other anions, the order of different cations is a bit different. With increasing *n*, the compressibility of [C_n_mim]^+^ increases, while that of [C_n_mpyr]^+^ decreases. On the other hand, the cation of [THTDP]^+^ always shows higher compressibility. The compressibilities of [C_n_mpy]^+^ and [C_n_py]^+^ are not related to the value of *n*. In contrast, according to the literatures, in the bulk phase, the physical CO_2_ solubility can be improved by increasing the alkyl chain length on the cation (Anthony et al., 2005). This indicates that, if the CO_2_ solubility is the only index for screening ILs, the ILs with the cation of long alkyl chain length will be selected. However, when the compressibility of ILs is considered, the screening result may be different.

According to the results shown in Figure 3 and Figure 4, and Supplementary Table S1, we can find that the compressibility of the ILs with [C_2_mpyr]^+^ (IL-cation) at 1 bar is from 0.31 to 0.68 Gpa^−1^, i.e., with a change of 0.37 Gpa^−1^, while the compressibility of ILs with the anion of [Tf_2_N]^-^ at 1 bar only changes from 0.46 to 0.66 Gpa^−1^, i.e., the difference is only 0.20 Gpa^−1^. This observation indicates that the influence of IL-anion on compressibility is more than that of IL-cation. Similar results can be observed for other cations and anions. Also, for the ILs with the same anion, their compressibility changes much less compared with the ILs with the same cation (Supplementary Figures S6–S14). Therefore, we can conclude that the compressibility of ILs is mainly affected by anion.

**FIGURE 5:**
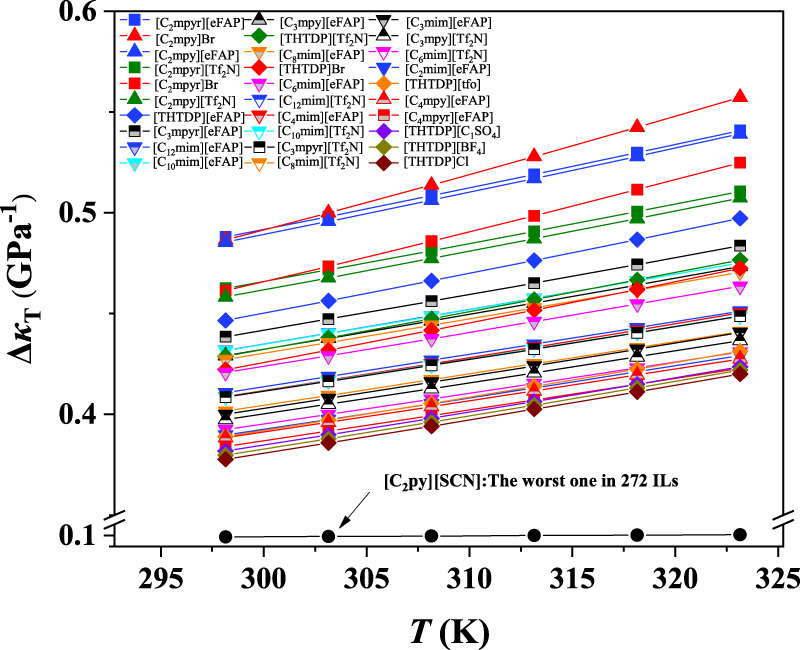
*Δκ*_T_ value of 30 ILs with better compresibility and the worst one.

According to Figure 1, the *κ*_T_ value for some ILs is not sensitive to the pressure, which makes it unobvious to perform screening. In order to differ the compressibility of ILs intuitively, the values of Δ*κ*_T_ (Δ*κ*_T_ *= κ*_T*,1*_ _bar_ *-κ*_T*,*6000_ _bar_) ranging from 298.15 to 323.15 K were calculated, and then ILs were screened. As depicted in Figure 5, 30 ILs with excellent compressibility are illustrated together with the worst one, i.e., [C_2_py][SCN]. With the increasing temperature, the Δ*κ*_T_ values of ILs increase, and ILs with the anion of Br^−^ are most sensitive to the temperature. Based on the screened 30 ILs, we observed that the ILs containing the cations of [C_2_mpyr]^+^, [C_2_mpy]^+^, [THTDP]^+^, [C_3_mpyr]^+^, [C_3_mpy]^+^, [C_12_mim]^+^, [C_10_mim]^+^ or the anions of [eFAP]^-^, [Tf_2_N]^-^ have better compressibility than other ILs.

### CO_2_ capacity

**FIGURE 6:**
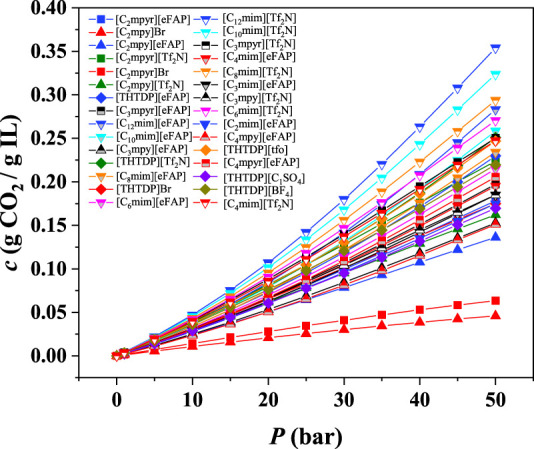
CO_2_ absorption capacity in 30 ILs at 298.15 K.

After the preliminary screening based on the compressibility, ePC-SAFT was also used to predict the CO_2_ absorption capacity for these 30 ILs. The results of CO_2_ absorption capacity are shown in Figure 6 and Supplementary Figures S15–S19 and listed in Supplementary Table S2.

As illustrated in Figure 6, the CO_2_ absorption capacity in ILs increases with increasing pressure. Most of ILs with the anion of [Tf_2_N]^-^ have better CO_2_ absorption capacity than the ILs with the anion of [eFAP]^-^. For the ILs with the cation of [C_n_mim]^+^, the CO_2_ absorption capacity increases with increasing the chain length. For example, the order of CO_2_ absorption capacity is in the order of [C_12_mim][Tf_2_N] > [C_10_mim][Tf_2_N] > [C_8_mim][Tf_2_N] > [C_6_mim][Tf_2_N] > [C_4_mim][Tf_2_N]. This is consistent with the experimental observations (Anthony et al., 2005), which also indicates that the results predicted by the ePC-SAFT model are reliable. Furthermore, not all ILs with high compressibility show good CO_2_ solubility. For example, the CO_2_ absorption capacities of [C_2_mpy]Br and [C_2_mpyr]Br are not as high as expected. Based on the results at 298.15 K, 7 ILs with higher CO_2_ absorption capacity were further screened, i.e., [C_12_mim][Tf_2_N], [C_10_mim][Tf_2_N], [C_8_mim][Tf_2_N], [C_12_mim][eFAP], [C_6_mim][Tf_2_N], [C_10_mim][eFAP], and [THTDP][Tf_2_N].

### Desorption enthalpy

**FIGURE 7:**
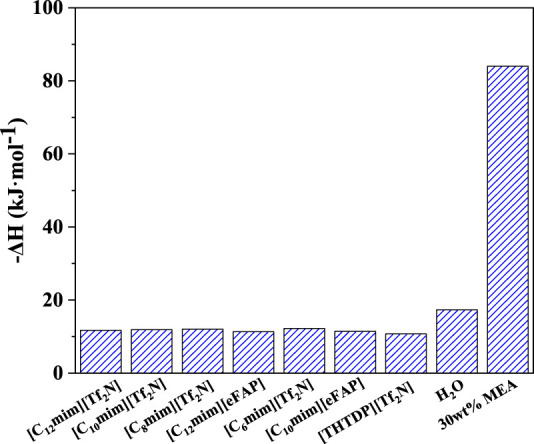
CO_2_ desorption enthalpy in 7 ILs compared with traditional solvents.

CO_2_ desorption enthalpy affects the energy demand in the desorption unit. H_2_O is the physical absorbent for biogas upgrading (i.e., CO_2_ removal), while 30 wt% monoethanolamine (MEA) is a chemical absorbent for CO_2_ separation from flue gases (Kim and Svendsen, 2011; Gupta et al., 2013; Chen et al., 2020). In this section, the CO_2_ desorption enthalpy of the above-screened 7 ILs was predicted, and the results were compared with the traditional solvents, as depicted in Figure 7 and listed in Supplementary Table S3. It can be seen that, among the screened ILs, [THTDP][Tf_2_N] has the lowest CO_2_ desorption enthalpy, and the ILs with the anion of [eFAP]^-^ show lower desorption enthalpy than that with the anion of [Tf_2_N]^-^. For example, the CO_2_ desorption enthalpy in [C_12_mim][eFAP] is lower than [C_12_mim][Tf_2_N], and the desorption enthalpy in [C_10_mim][eFAP] is also lower than [C_10_mim][Tf_2_N]. Anyhow, the desorption enthalpy of all the screened ILs is around −12 kJ/mol, confirming the physical interaction between CO_2_ and the selected ILs. Therefore, CO_2_ desorption enthalpy can be out of consideration when screening the physical ILs. Compared with H_2_O and 30 wt% MEA, the desorption enthalpy of the screened ILs is approximately reduced by 30 and 85%, respectively.

### Verification

**FIGURE 8:**
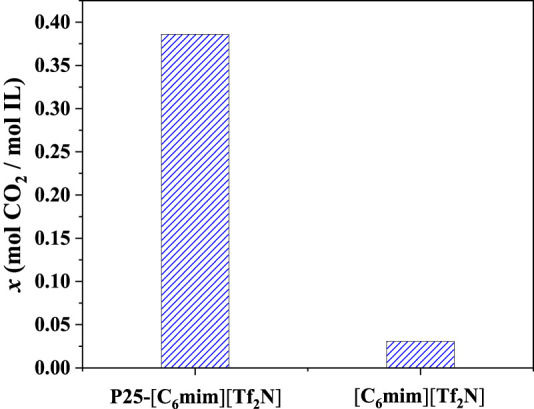
CO_2_ absorption capacity of P25-[C_6_mim][Tf_2_N] and [C_6_mim][Tf_2_N].

**FIGURE 9:**
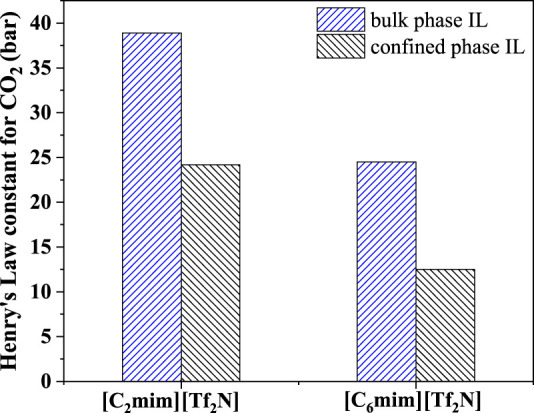
Henry’s constants of CO_2_ in [C_2_mim][Tf_2_N] and [C_6_mim][Tf_2_N].

Among the screened 7 ILs, [C_6_mim][Tf_2_N] immobilized on the surface of titanium dioxide with a particle size of 25 nm (P25) has been studied in the previous study (Wu et al., 2017), showing desirable CO_2_ separation performance. As shown in Figure 8, the CO_2_ absorption capacity of [C_6_mim][Tf_2_N]/P25 is about ten times that in the bulk [C_6_mim][Tf_2_N] at 298.15 K and atmospheric pressure. Banu et al. (Banu et al., 2013) studied CO_2_ in the [C_2_mim][Tf_2_N] and [C_6_mim][Tf_2_N] confined in the ceramic porous membrane at 298.15 K and atmospheric pressure. Figure 9 shows that after immobilization, the CO_2_ absorption capacities in [C_2_mim][Tf_2_N] and [C_6_mim][Tf_2_N] were enhanced by about 1.6 and 2 times, respectively, and the CO_2_ absorption capacity intensification in [C_6_mim][Tf_2_N] is higher than that in [C_2_mim][Tf_2_N]. According to the result in Figure 4, the compressibility of [C_6_mim][Tf_2_N] is much higher than that of [C_2_mim][Tf_2_N], which is consistent with the trend of the CO_2_ absorption capacity. Therefore, it can be concluded that the index proposed in this work is reasonable, and the compressibility of ILs is an important index in screening ILs for developing IL-immobilized absorbent for CO_2_ separation.

As only limited experimental results on the immobilized ILs are available, in the future, the CO_2_ separation performance of IL-immobilized absorbents will be determined experimentally to systematically verify the screen method and results. In addition, the CO_2_ separation performance is a combination of thermodynamics and kinetics, and some ILs can absorb CO_2_ chemically. In the future, the research on the screen will be extended to include CO_2_ chemical absorption and consider the kinetic contribution to the CO_2_ separation performance.

## Conclusion

In this study, a new additional index, i.e., the compressibility of ILs, was proposed to screen ILs for developing IL-immobilization technology for CO_2_ separation. The developed ePC-SAFT model was used as a theoretical tool to predict reliable and systematic data for screening. From 272 physical ILs, 30 ILs were selected firstly based on the compressibility. Then, 7 ILs, i.e., [C_12_mim][Tf_2_N], [C_10_mim][Tf_2_N], [C_8_mim][Tf_2_N], [C_6_mim][Tf_2_N], [C_12_mim][eFAP], [C_10_mim][eFAP], and [THTDP][Tf_2_N], were finally screened based on the CO_2_ absorption capacity and desorption enthalpy. Finally, the CO_2_ separation performance with [C_6_mim][Tf_2_N] was compared with the experimental results and discussed, verifying the reliability of the IL screening. In the future, systematic experiments will be designed to further verify this screen index, and the quantitative relationship between compressibility and CO_2_ absorption capacity will be studied to further understand the mechanism of the enhanced CO_2_ absorption capacity in the immobilized ILs.

## Data Availability

The original contributions presented in the study are included in the article/Supplementary Material, further inquiries can be directed to the corresponding authors.

## Nomenclature

### Abbreviations

ILs: Ionic liquids
ePC-SAFT: Electrolyte perturbed-chain statistical associating fluid theory
**[C**_**n**_ **mim]** ^**+**^: 1-n-alkyl-3-methylimidazolium cation
**[C**_**n**_ **py]** ^**+**^: 1-n-alkyl-pyridinium cation
**[C**_**n**_ **mpy]** ^**+**^: 1-n-alkyl-3-methylpyridinium cation
**[C**_**n**_ **mpyr]** ^**+**^: 1-methyl-1-n-alkylpyrrolidinium cation
**[THTDP]** ^**+**^: Trihexyltetradecylphosphonium cation
**[Tf**_**2**_ **N]** ^**-**^: Bis(trifluoromethyl-sulfonyl)amide anion
**[PF**_**6**_]^**-**^: Hexafluorophosphate anion
**[BF**_**4**_ **]** ^**-**^: Tetrafluoroborate anion
**[tfo]** ^**-**^: Trifluoromethansulfonate anion
**[DCA]** ^**-**^: Dicyanamide anion
**[SCN]** ^**-**^: Thiocyanate anion
**[C**_**1**_ **SO**_**4**_ **]** ^**-**^: Methylsulfate anion
**[C**_**2**_ **SO**_**4**_ **]** ^**-**^: Ethylsulfate anion
**[eFAP]** ^**-**^: Tris(pentafluoroethyl)trifluorophosphate anion
**Cl** ^**−**^: Chloride anion
**[Ac]** ^**-**^: Acetate anion
**Br** ^−^: Bromide anion

### Symbols

***κ***_***T***_: Isothermal Compression Coefficient
***ΔH***: Enthalpy
***P***: Pressure
***T***: Temperature
***A***: Helmholtz energy
***κ***: Inverse Debye-screening length
ε: _τ_ Relative dielectric constant of the medium: _0_ Dielectric constant of vacuum
ε: _τ_ Relative dielectric constant of the medium: _0_ Dielectric constant of vacuum
$x_{j}$: Mole fraction of ion j
$q_{j}$: Charge of ion j
ρ: Density
$\varphi_{CO_{2}}^{L}$: Fugacity coefficients for CO_2_ in the liquid phases
$\varphi_{CO_{2}}^{V}$: Fugacity coefficients for CO_2_ in the vapor phases
$v^{L}$: Molar volumes of liquid phases
$v^{V}$: Molar volumes of vapor phases
$H_{CO_{2}}$: Henry coefficient of CO_2_
***R***: Gas constant
***Z***: Compressibility factor
***P***_**c**_: Critical pressure
***T***_**c**_: Critical temperature
***k***: Boltzmann constant
$\mu_{CO_{2}}^{res}\left(T,v^{V}\right)$: Chemical potential of CO_2_
