# Supplementary material for: Screening ionic liquids for developing advanced immobilization technology for CO2 separation
Source: Front Chem. 2022 Jul 12;10:941352. doi: 10.3389/fchem.2022.941352 (PMC9321636; doi:10.3389/fchem.2022.941352)
Supplement: Supplementary file 1 [file DataSheet2.docx]

Supplementary Material

# Supplementary Figures

Fig. S1. *κ*_T_ of 272 ILs at 303.15 K.

Fig. S2. *κ*_T_ of 272 ILs at 308.15 K.

Fig. S3. *κ*_T_ of 272 ILs at 313.15 K.

Fig. S4. *κ*_T_ of 272 ILs at 318.15 K.

Fig. S5. *κ*_T_ of 272 ILs at 323.15 K.

Fig. S6. *κ*_T_ value of ILs with [C_2_py]^+^.

Fig. S7. *κ*_T_ value of ILs with [C_2_mpy]^+^.

Fig. S8. *κ*_T_ value of ILs with [C_2_mim]^+^.

Fig. S9. *κ*_T_ value of ILs with [THTDP]^+^.

Fig. S10. *κ*_T_ value of ILs with [eFAP]^-^.

Fig. S11. *κ*_T_ value of ILs with [C_2_SO_4_]^-^.

Fig. S12. *κ*_T_ value of ILs with [BF_4_]^-^.

Fig. S13. *κ*_T_ value of ILs with [PF_6_]^-^.

Fig. S14. *κ*_T_ value of ILs with [SCN]^-^.

Fig. S15. CO_2_ absorption capacity in 30 ILs at 303.15 K.

Fig. S16. CO_2_ absorption capacity in 30 ILs at 308.15 K.

Fig. S17. CO_2_ absorption capacity in 30 ILs at 313.15 K.

Fig. S18. CO_2_ absorption capacity in 30 ILs at 318.15 K.

Fig. S19. CO_2_ absorption capacity in 30 ILs at 323.15 K.
